# Kinetic energy management in road traffic injury prevention: a call for action

**DOI:** 10.5249/jivr.v7i1.458

**Published:** 2015-01

**Authors:** Davoud Khorasani-Zavareh, Maryam Bigdeli, Soheil Saadat, Reza Mohammadi

**Affiliations:** ^*a*^Social Determinant of Health Research Center, Urmia University of Medical Sciences, Urmia, Iran.; ^*b*^Department of Clinical Science and Education, Södersjukhuset, Karolinska Institutet, Stockholm, Sweden.; ^*c*^Sina Trauma and Surgery Research Centre, Tehran University of Medical Sciences, Tehran, Iran.; ^*d*^Division of Social Medicine, Department of Public Health Sciences, Karolinska Institutet, Stockholm, Sweden.

**Keywords:** Kinetic energy, Road traffic, Injury, Prevention

## Abstract

By virtue of their variability, mass and speed have important roles in transferring energies during a crash incidence (kinetic energy). The sum of kinetic energy is important in determining an injury severity and that is equal to one half of the vehicle mass multiplied by the square of the vehicle speed. To meet the Vision Zero policy (a traffic safety policy) prevention activities should be focused on vehicle speed management. Understanding the role of kinetic energy will help to develop measures to reduce the generation, distribution, and effects of this energy during a road traffic crash. Road traffic injury preventive activities necessitate Kinetic energy management to improve road user safety.

## Introduction

In the concept of road traffic injury (RTI), mass and speed are properties of all the energy that can be transferred during a crash; and the two properties are connected to kinetic (mechanical) energy. The amount of energy interchange can result injury severity that is equal to one half of the vehicle mass multiplied by the square of the vehicle speed. ^1^ This means that the kinetic energy during a collision greatly increases due to velocity rather than mass and consequently, small increases in vehicle speed will result in major increases in the risk of injury. ^2^ In order to have better insight into the amount of energy involved, it should be considered that one Joule is approximately the energy released when a textbook is dropped on the floor. Speed is a well known key risk factor in the risk of crash occurrence as well as in the severity of an injury. ^3^ For example, kinetic energy with a motorcycle weighing 150 kg and traveling at 60 km/h speed can produce 270,000 joule, while with a speed 120 km/h, the same motorcycle can produce 1,080,000 joule. This energy can be transferred in a fraction of second and is the reason why an increase in speed can be harmful or fatal. Despite speed playing the most critical role in RTIs, the extent of bodily injury, however, also depends on the shape of the objects involved and their rigidity as well as on what safety equipment is available. ^2^ This is why kinetic energy management needs to focus more on speed, which can be an important implication in RTIs prevention. 

If a pedestrian is hit by a vehicle traveling at less than 30 km/h, the risk of bodily injury will be less than 10%, but this rises to about 50% if the vehicle is traveling at 45 km/h. ^1^ The safest vehicles can provide injury protection to car riders in frontal collisions at around 70 km/h and in side-impact collisions at around 50 km/h for those wearing seat belts. ^1^ This may be why in Vision Zero, a traffic safety policy in RTIs prevention that introduced in Sweden, when implementation of safe transport system deal with challenges, all activities should focus on speed management. This is especially true in low and middle-income countries (LMICs). According to Vision Zero, in situations where there is possible conflict between pedestrians and cars, the speed should be limited to 30 km/h; and in situations where there is a risk of side-impact vehicle collisions, it should be limited to 50 km/h. As a matter of fact, when driving at 10 km/h, the vehicle travels 2.8 m every second; at 50 km/h it travels 14 m/s; and at 100 km/h it travels at 28 m/s. Just two second negligence by the driver can result in the vehicle traveling 56 meters further. Although the braking distance depends on the speed and mass of vehicle and its braking proficiency, most vehicles traveling in a dry pavement need 36 m, 45 m and 57 m to stop when traveling with a speed of 50, 60 and 70 km/h respectively, taking into account the driver reaction time. This means that a pedestrian appearing 45 m ahead of a car traveling with a speed of 70 km/h will be hit on a speed of 46 km/h, while the impact could be avoided if the car had been traveling with a speed of less than 60 km/h. ^4^ In many LMICs, however, there is still not enough attention paid to speed limits in urban areas. 

According to Vision Zero "…the speed limits within the road transport system should be determined by the technical standard of vehicles and roads so as not to exceed the level of violence that the human body can tolerate. The safer the roads and vehicles, the higher the speed that can be accepted". ^5^ Realizing a safe transport system is predictably a big challenge in many LMICs. With regard to of Vision Zero, however, it is clear that most road user safety measures should focus on speed management. 

Taking a system approach to RTIs prevention,^6^ harmful road traffic injury is in fact the result of ‘energy interchange’.^1,3^ Understanding the role of kinetic energy is important for all stakeholders in road traffic injury preventive activities. This will help them to develop measures to decrease the generation, distribution, transfer and effect of this energy during a road traffic crash.^1^ In conclusion, in order to improve road user safety and considering the wide array of vehicle types in most LMICs, “kinetic energy management” needs to be considered in all road traffic injury preventive activities.^6^
